# Valorization of Olive Pruning Residues through Bioconversion into Edible Mushroom *Pleurotus ostreatus* (Jacq. Ex Fr.) P. Kumm. (1871) of Improved Nutritional Value

**DOI:** 10.1155/2020/3950357

**Published:** 2020-07-14

**Authors:** Sami Abou Fayssal, Mohammed A. Alsanad, Zeina El Sebaaly, Ahmed I. H. Ismail, Youssef N. Sassine

**Affiliations:** ^1^University of Forestry, 10 Kliment Ohridski Blvd, BG1797 Sofia, Bulgaria; ^2^Department of Plant Production, Faculty of Agriculture, Lebanese University, Beirut, Lebanon; ^3^Department of Environment and Agricultural Natural Resources, College of Agricultural and Food Sciences, King Faisal University, P.O. Box 400, Al Ahsa 31982, Saudi Arabia; ^4^Agribusiness and Consumer Sciences Department, College of Agriculture and Food Sciences, King Faisal University, P.O. Box 420, Al-Ahsa 31982, Al-Hofuf, Saudi Arabia; ^5^Rural Community and Agric. Extension Department, College of Agriculture, Ain Shams University, Cairo, Egypt; ^6^Department of Agricultural Biotechnology, College of Agricultural and Food Sciences, King Faisal University, P.O. Box 420, Al Ahsa 31982, Saudi Arabia

## Abstract

In several Mediterranean countries, olive pruning residues (OLPRs) are abandoned or burned leading to several environmental problems. Valorization of these agrowastes could be a challenge for the primary decomposer *Pleurotus ostreatus*, turning them into edible biomass. The OLPR was used alone (OLPR), or in mixtures with wheat straw (WS : OLPR 1 : 3 v/v and WS : OLPR 3 : 1 v/v). Mycelial colonization was hastened by 3.7 days in WS : OLPR 1 : 3 (v/v). Yields were comparable to control (WS) in WS : OLPR 3 : 1 (v/v). Organic matter loss decreased with increasing proportions of OLPR in substrates. The nutritional value of mushrooms was improved by lower fat and sodium contents, in WS : OLPR 1 : 3 (v/v) and WS : OLPR 3 : 1 (v/v), and higher total protein, crude fiber, iron, and total carbohydrates contents in WS : OLPR 3 : 1 (v/v), compared with those of control. Polyunsaturated fatty acids, mainly linoleic acid, were the most abundant in mushrooms. Monounsaturated fatty acids increased in mushrooms of the substrates containing OLPR. A good predictive model of partial least square regression analysis showed different relationships of mushroom palmitic, oleic, linolenic, palmitoleic, and stearic acids with substrate composition. Findings suggested the use of OLPR as a supplement to commercial wheat straw and as a tool to reduce the negative impacts of their hazardous disposal on the environment.

## 1. Introduction

Mushrooms of the genus *Pleurotus*, otherwise known as oyster mushrooms, rank second in the world mushroom market and are the most popular in China [1]. *Pleurotus ostreatus* (Jacq.). P. Kumm. 1871 is particularly treasured compared with other species of the same genus because of its delicious taste, richness in proteins, carbohydrates, minerals (calcium, phosphorus, and iron) and vitamins (thiamine, riboflavin, and niacin). It is low in fat [2] and contains some essential fatty acids with dominance for the unsaturated ones [3]. The mushroom possesses nutritional qualities especially valued in vegetarian [4] and calorie-controlled diets, because of its low calorific value [5].

Moreover, *P. ostreatus* has considerable economic value because of its flexible nature allowing it to grow on a variety of agricultural wastes [6] due to its exceptional ligninolytic properties [7, 8]. It is a saprophyte [9] that requires carbon, nitrogen, and inorganic compounds for nutrition [10].

As mushrooms grow by bioconversion of agricultural wastes [11], their nutritional value largely depends on the chemical composition of the substrate [12–14]. Besides chemical, functional, and sensorial characteristics of mushrooms [15, 16], the substrate type will affect yield and biological efficiency [10]. Therefore, it is essential to know the chemical composition of the substrate before its use in mushroom cultivation [17]. In commercial production, cereal straw (mainly wheat straw) is commonly used as substrate for *P. ostreatus* [18], although several agricultural by-products, such as maize wastewater [19] and olive mill wastes [20] were suitable for mushroom production. Large-scale cultivation requires a good knowledge of the substrate influence on the mushroom nutritional composition [21], especially that the characteristics and composition of the agricultural waste are subject to a wide variation [22], and depend on the system and type of agricultural activities [23]. Nowadays, agrowastes have become a tremendous natural challenge, and their incorporation in mushroom production has been investigated [24]. Immense biomass potential, in the form of olive tree pruning residues, is annually generated and abandoned. Most of these residues are burned by farmers in open-field, generating dioxins and other pollutants and causing environmental hazards [25, 26]. Olive tree (*Olea europaea*) is the most economically important oil-producing crop in many Mediterranean countries [27]. From 750 million olive trees cultivated worldwide, 95% grow in the Mediterranean region [28]. Olive tree cultivation covers 23% of the total agricultural land in Lebanon [29], generating annually huge amounts of plant residues or pruning wastes. The mismanagement of agricultural wastes generated by olive tree cultivation has been the reason behind the present study, which focused mainly on pruning residues. It consisted of a trial to incorporate those residues, in different proportions, in the growing substrate of *P. ostreatus* mushroom and therefore to test their effect on mushroom production and nutritional value.

## 2. Materials and Methods

### 2.1. Experimental Treatments

The experiment investigated the effect of four substrates on mushroom production and nutritional composition. In particular, the first substrate was wheat straw (WS) or control substrate, the second was olive pruning residues (OLPR), and the third and fourth substrates were a mixture of WS and OLPR, done on volume basis: WS : OLPR 1 : 3 (v/v) and WS : OLPR 3 : 1 (v/v). A complete randomized design was adopted with four treatments (substrates) and ten replicates (10 bags) per treatment.

### 2.2. Substrate Preparation, Spawning, and Incubation

Wheat straw and olive pruning residues (OLPR) were procured from a local private enterprise (Compost Baladi) in fresh state. Olive pruning residues consisted of a mixture of thin woody sticks and leaves left after pruning, fermented outdoor for one year before use. Both tested substrates were sun-dried for two days prior to use. They were then pasteurized at 60–65°C with boiling water for eight hours and cooled down to the spawning temperature (25°C) [30].

Cereal grain spawn of *P. ostreatus* (strain M 2175) was prepared in glass jars at the laboratory of Food Technology of the Faculty of Agricultural Engineering and Veterinary Medicine. Spawn was added with a rate of 5% w/w to the substrate and 50 g of gypsum CaCO_3_ (2% w/w in terms of dry weight) was added in order to adjust the substrate pH. Inoculated substrates were filled into perforated transparent polyethylene bags (60 cm length × 40 cm width). Holes were evenly made on the sides of the bags, which were placed in a cropping chamber in dark conditions at 23–25°C and 85–90% relative humidity until complete mycelial colonization. Fourteen days after incubation, the stage of pinhead formation was then stimulated by lighting, reduction of the room temperature to around 16°C, and ventilation to keep CO_2_ levels below 900–2300 ppm.

### 2.3. Evaluation of Mushroom Production and Quality

Prior to filling, squares of 5 × 5 cm were drawn on each bag. The time to full mycelial colonization (100% MC) was recorded when all squares became white. The time to harvest of first flush (HF1) was determined as number of days after spawning (DAS). For every treatment, the number of mushroom flushes, biological yield (g/bag), and economic yield (g/bag) were assessed. Economic yield (g/bag) corresponded to the total weight of effective fruit bodies after removal of the base of stalks [31]. In addition, the biological efficiency (BE) and organic matter loss (OML) were calculated [32] as follows:

(1) $$\begin{array}{c} \text{BE}\left(\text{\%}\right)=\frac{\text{total fresh mass of mushrooms}\left(\text{g}\right)}{\text{initial dry mass of substrate}\left(\text{g}\right)}\times \;100,\\ \text{OML}\left(\%\right)=\frac{\text{initial dry mass of substrate}\left(\text{g}\right)-\text{residual}\left(\text{g}\right)}{\text{initial dry mass of substrate}\left(\text{g}\right)}\times 100. \end{array}$$

Using ten representative samples selected per replicate (bag) relative to each treatment, physical characteristics of mushrooms were evaluated by measuring mushroom weight (g), pileus diameter (cm), stipe diameter (cm), and stipe length (cm) and by determining the ratio of pileus diameter over stipe length (PD/SL) as a qualitative indicator.

### 2.4. Analysis of Substrate Properties

Analyzed substrate properties (Table 1), included pH using a pH meter (UltraBasic-UB10; Denver Instrument), electroconductivity using an EC meter (SC-2300 conductivity meter; Suntex Instrument), total organic matter content (via loss of ignition method at 430°C over 24 h), moisture content (using moisture analyzer, Sartorius Instrument, Model MA37), and C/N ratio using a CHN analyzer with an automatic sampler (Carlo-Erba elemental analyzer, Model 1106).

Mineral composition of substrates (K, Ca, Mg, Na, Fe, and Mn) was analyzed using atomic absorption spectrophotometry following AOAC [33] standards. The determination of total protein content was carried out using the micro-Kjeldahl method (*N* × 6.25) [33]. Fat content was determined by continuous extraction using the Soxhlet apparatus [33]. An enzymatic gravimetric method was applied to determine the crude fiber content in the samples according to AOAC [34]. Total carbohydrates were determined using the anthrone method. Total sugars and sugar composition including fructose, glucose, and sucrose contents were analyzed using high-performance liquid chromatography (HPLC) as described by Ajlouni et al. [35]. Fatty acid composition was determined using gas chromatography-mass spectrometry as described by Nieto and Chegwin [36]. All tests of substrate composition were performed in analytical triplicates. Furthermore, fiber fractions of residual substrates, cellulose, hemicellulose, lignin, neutral detergent fiber (NDF), acid detergent fiber (ADF), and acid detergent lignin (ADL) were analyzed on dry samples using the ANKOM technology method, filter bag technique (08-16-06, 08-05) following the AOAC official method of analysis [34, 37].

### 2.5. Analysis of Mushroom Composition

Mushroom composition was analyzed using fresh samples of mushroom pileus. Mushroom samples were analyzed for fat and crude fiber contents using the AOAC [37] procedures. Fat content was determined by extracting a known weight of powdered sample with ethyl ether, using a Soxhlet apparatus. Determination of total carbohydrates content followed the anthrone method [38]. Total protein content of samples was estimated by the macro-Kjeldahl method (*N* × 4.38) [39]. Mineral contents (Ca, Mg, Fe, Mn, K, and Na) were analyzed by ICP atomic emission spectrophotometry after element extraction in 0.1 N HCl acidic solution. The fatty acid profile was determined by gas chromatography (Agilent 6890 gas chromatography (Palo Alto, CA) following the method in [40]). Total soluble sugars, fructose, glucose, and sucrose contents were analyzed using high-performance liquid chromatography (HPLC). Moisture content was determined using a moisture analyzer (M5-Thermo A64M). All tests of mushroom composition were performed in five analytical replicates relative to each experimental replicate.

### 2.6. Statistical Analysis

Using the program Statistical Package for Social Sciences SPSS 25®, one-way ANOVA and Duncan tests were applied for data analysis. Pearson's correlations were established between substrates and mushroom composition. A simple regression was performed to test the relation between organic matter loss (dependent variable) and OLPR proportion in the substrate (predictors). Additionally, partial least squares regression (PLSR) was applied for multivariate analysis, using XLSTAT statistical and data analysis solution, Addinsoft 2019, Boston, MA, USA. The Jackknife (LOO) test was used for cross-validation of the resulting models. Confidence levels of 95% or 99% were adopted for statistical tests.

## 3. Results

### 3.1. Effect of Substrate on Mushroom Production

Results of one-way ANOVA (analysis of variance) (Table 2) showed that the substrate effect was statistically (*p* < 0.05) significant on averages of biological yield, number of flushes, and time to harvest the first flush, while it was not significant on time to complete substrate colonization, biological efficiency, and mushroom weight.

The substrate OLPR was not completely colonized by the mycelium; therefore, it was not productive, and it was excluded from the results part. In comparison with control, mushroom production was hastened by 3.7 days and delayed by 11.3 days in the substrates WS : OLPR 1 : 3 (v/v) and WS : OLPR 3 : 1 (v/v), respectively. In addition, there was a significant reduction in the average number of flushes in substrates containing OLPR. Though in the substrate WS : OLPR 3 : 1 (v/v), averages of biological yield, biological efficiency, and economic yield were comparable to control, and these indicators were significantly reduced in the substrate WS : OLPR 1 : 3 (v/v).

**Figure 1:**
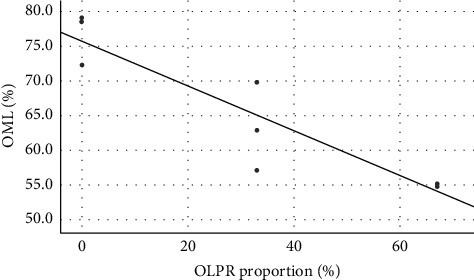
Relation between organic matter loss (OML) and proportions of olive pruning residues (OLPRs) (OML = −0.32 × OLPR + 75.76) (*r*^*2*^ = 0.85).

Moreover, results of the regression analysis (Figure 1) showed a strong linear relationship (*r*^*2*^ = 0.85) between the proportion of OLPR and organic matter loss in the substrates, which reflects a gradual decrease in means of the latter with increasing values of the former.

### 3.2. Analysis of Residual Substrates

The three lignocellulosic components (Table 3), cellulose, hemicellulose, and lignin, were initially different in the tested substrates. Hemicellulose and cellulose were more abundant in wheat straw than in substrates containing OLPR, while lignin was more abundant in the latter substrates. Hemicellulose was most reduced in the wheat straw substrate (reduction by 97.2% compared with 82.0 and 49.7% in substrates WS : OLPR 3 : 1 (v/v) and WS : OLPR 1 : 3 (v/v), respectively), while cellulose was the most reduced in the substrate WS : OLPR 3 : 1 (v/v) (reduction by 47.8% compared with 33.4 and 43.6% in wheat straw and WS : OLPR 1 : 3 (v/v), respectively). Additionally, reduction in lignin content was higher in the wheat straw substrate than in substrates WS : OLPR 3 : 1 (v/v) and WS : OLPR 1 : 3 (v/v) (53.6% compared with 3.33 and 30.9%, respectively).

### 3.3. Appearance and Nutritional Value of Mushroom

**Figure 2:**
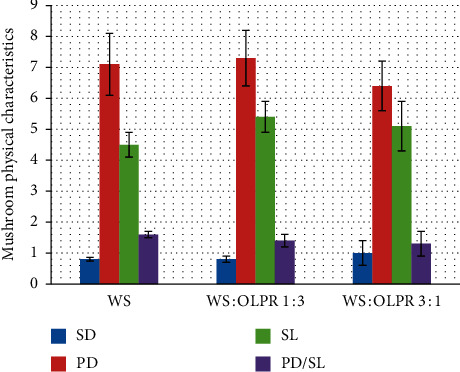
Physical characteristics of mushrooms obtained in tested substrates (SD: stipe diameter, PD: pileus diameter, SL: stipe length, PD/SL: pileus diameter/stipe length, and ns: no significance).

The effect of the substrate was not significant on mushroom's physical appearance; pileus diameter (*p*=0.46), stipe diameter (*p*=0.48), stipe length (*p*=0.24), and PD/SL ratio (*p*=0.47). Stipe diameter ranged between 0.8 and 1.0 cm, pileus diameter between 6.4 and 7.3 cm, stipe length between 4.5 and 5.4 cm, and PD/SL ratio between 1.3 and 1.6 (Figure 2).

Results in Table 4 show that substrate moisture content increased by 7.2%, and 15.9%, with WS : OLPR 3 : 1 (v/v) and WS : OLPR 1 : 3 (v/v) compared with control, while it did not differ in all harvested mushrooms. Total protein content increased by 1.8% in the substrate WS : OLPR 1 : 3 (v/v) and by 0.8% in mushrooms of the substrate WS : OLPR 3 : 1 (v/v), in comparison with control. The substrate WS : OLPR 1 : 3 (v/v) was the richest in crude fiber and carbohydrates. Crude fiber content was improved by 1.4% in mushrooms produced by the substrate WS : OLPR 3 : 1 (v/v). Fat content was reduced in mushrooms produced in all substrates containing OLPR in comparison with control mushrooms. Control substrate was the richest in total soluble sugars, including fructose, glucose, and sucrose, compared with remaining substrates. Among the three sugars, glucose was the most abundant in all tested substrates. All mushrooms had very low sucrose content compared with glucose and fructose contents. Correlations of indicators between substrates and mushrooms showed strong negative interrelationships at *p*_value_ = 0.01 in terms of total protein (*r* = –0.93), crude fiber (*r* = –0.81), carbohydrates (*r* = –0.99), total soluble sugars (*r* = –0.97), and glucose (*r* = –0.91).

Analysis of mineral composition (Table 5) showed that calcium content was similar in mushrooms although it was different in tested substrates. The incorporation of OLPR has caused a decrease in the potassium content of substrates. Manganese and magnesium contents were not significantly different in all mushrooms, although the former was lower and the latter was higher in substrates containing OLPR compared with that in control. Sodium content was lower in substrates containing OLPR and mushrooms produced in comparison with control substrate and control mushrooms. Those substrates had significantly higher iron content than that of the control substrate, which explains the high iron content in mushrooms. Correlations of indicators between substrates and mushrooms showed a strong negative interrelationship at *p*_value_ = 0.05 in terms of potassium (*r* = –0.73) and a strong positive interrelationship at *p*_value_ = 0.01 in terms of sodium (*r* = 0.81).

### 3.4. Changes in the Fatty Acid Profile of Mushrooms

The analysis of the fatty acid profile of substrates (Table 6) showed that palmitic acid was the most abundant in the substrates WS and WS : OLPR 3 : 1 (v/v), and linoleic acid was the most abundant in the substrate WS : OLPR 1 : 3 (v/v). Linoleic acid was higher in mushrooms than in substrates and the highest in control mushrooms. Palmitoleic acid was not detected in both substrates containing OLPR but was found in mushrooms produced by these substrates. Myristic acid, absent in all substrate types, was found in mushrooms of WS : OLPR 1 : 3 (v/v) and WS : OLPR 3 : 1 (v/v). Mushrooms obtained in control substrate contained around 80.8% polyunsaturated fatty acids, while those obtained in WS : OLPR 1 : 3 (v/v) and WS : OLPR 3 : 1 (v/v) contained around 64.2% and 69.0%, respectively. Oleic acid increased by 5.6% in mushrooms of the substrate WS : OLPR 1 : 3 (v/v) compared with that of control. Arachidic acid present in the substrates containing OLPR was absent in mushrooms of these substrates. Mushrooms produced in the substrates containing OLPR were richer in monounsaturated fatty acids compared with control mushrooms.

Furthermore, partial least squares regression (PLSR) analysis was used to define the possible interrelationships between chemical composition of substrates (minerals, fatty acids, total protein, carbohydrates, fat, and crude fiber) as independent variables (represented by the *X*-matrix) and mushroom fatty acids as dependent ones (represented by the *Y*-matrix). The coefficient *r*^*2*^ between *Y* and (*t*1, *t*2) gives an upper bound of how well the model explains the data and predicts new observations. The cross-validation *q*^*2*^ cum defines the stability of the model and sets the lower bound of how well the model explains the data. In addition, the root-mean-squared error (*RMSE*) measures the difference between observed and predicted values of a model. It was suggested that a smaller value of *RMSE* indicates that the model fits better the experimental data [41]. *RMSE* was low for the built model (Table 7), indicating a reliable one and displaying a good prediction of *Y* values using *X* values.

**Figure 3:**
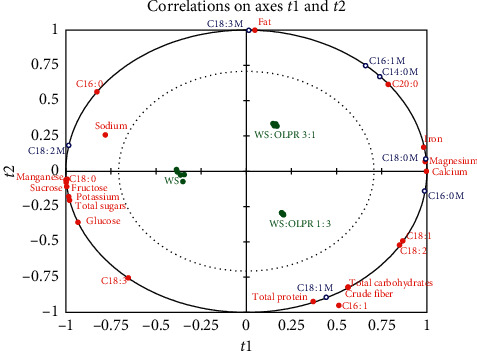
Correlations between mushroom fatty acids and substrates chemical composition: C14 : 0: myristic acid, C16 : 0: palmitic acid, C16 : 1: palmitoleic acid, C18 : 0: stearic acid, C18 : 1: oleic acid, C18 : 2: linoleic acid, C18 : 3: linolenic acid, C20 : 0: arachidic acid, and M: mushroom.

**Figure 4:**
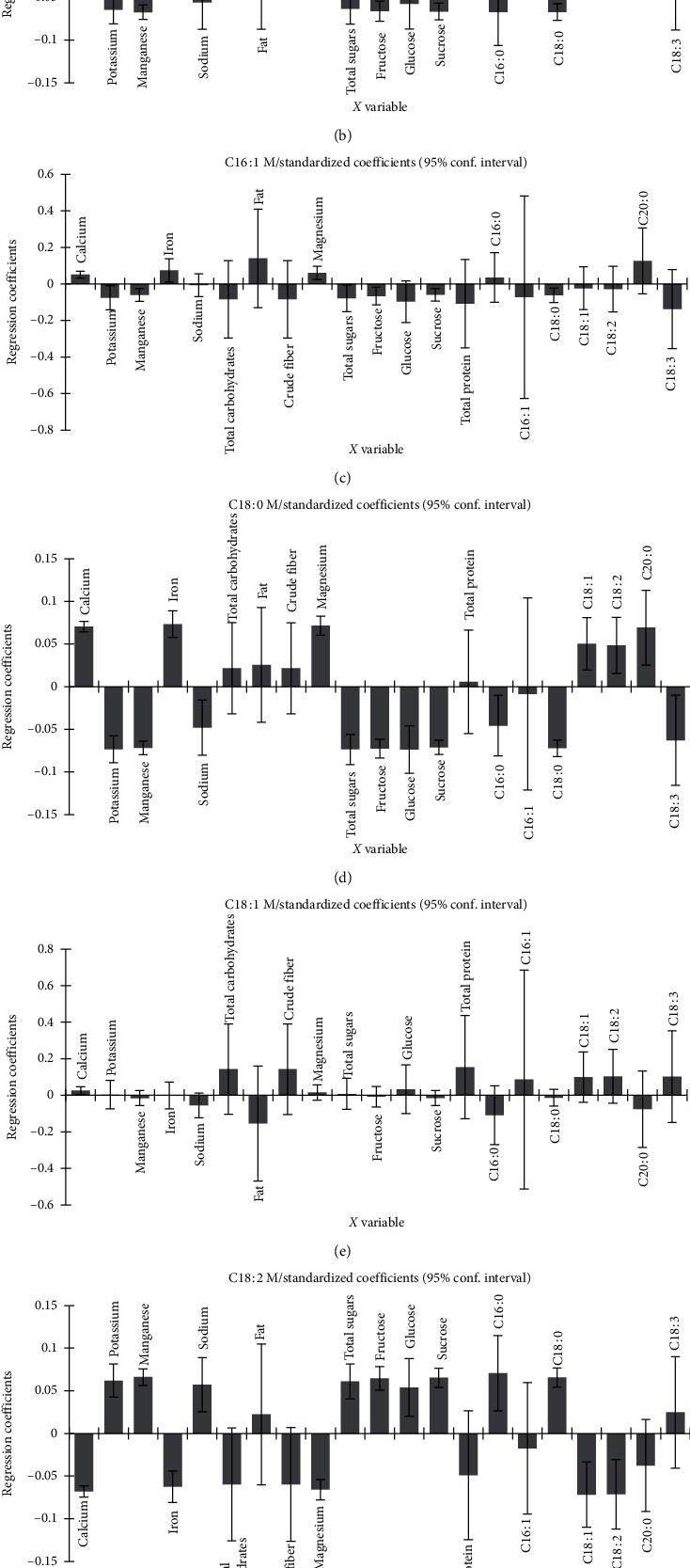
Regression coefficients and significant indications (shown in streaked bars) for substrate chemical composition variable: C16 : 0: palmitic acid, C16 : 1: palmitoleic acid, C18 : 0: stearic acid, C18 : 1: oleic acid, C18 : 2: linoleic acid, C18 : 3: linolenic acid, C20 : 0: arachidic acid, and M: mushroom. (a) C14 : 0 M/standardized coefficients (95% conf. interval). (b) C16 : 0 M/standardized coefficients (95% conf. interval). (c) C16 : 1 M/standardized coefficients (95% conf. interval). (d) C18 : 0 M/standardized coefficients (95% conf. interval). (e) C18 : 1 M/standardized coefficients (95% conf. interval). (f) C18 : 2 M/standardized coefficients (95% conf. interval). (g) C18 : 3 M/standardized coefficients (95% conf. interval).

Correlations on axes *t*1 and *t*2 between mushroom fatty acids and substrates chemical composition showed two components for *X* variables and two components for *Y* variables. In addition, only component 1 was enough to predict correlations between palmitic, stearic, and linoleic acids with substrate composition. The presence of *X* variables (except palmitoleic acid) and *Y* variables between the inner and outer ellipses (Figure 3) indicates that correlations between these variables can be well explained by the relative PLSR model. Furthermore, regression coefficients (Figures 4(a)–4(g)) were calculated in order to determine significant contribution of *X* variable(s) to variation of relative *Y* variable(s).

The predictive model (Figure 3) delineated a covariation of mushroom myristic and palmitoleic acids with fat, arachidic acid, iron, magnesium, and calcium contents in the substrate WS : OLPR 3 : 1 (v/v). According to the regression variation of both, mushroom fatty acid was the most positively correlated to fat and arachidic acid contents and negatively correlated with linolenic acid content of substrates (Figures 4(a) and 4(c)). Mushroom palmitic acid covaried with magnesium, calcium, iron, total protein, total carbohydrates, crude fiber, oleic acid, and linoleic acid contents, in the substrate WS : OLPR 1 : 3 (v/v) (Figure 3). However, variation in this acid was most strongly correlated with oleic and linoleic acids, iron, calcium, and magnesium contents, while it was most negatively correlated with manganese and palmitic and stearic acid contents (Figure 4(b)). Mushroom stearic acid covaried with fat, magnesium, calcium, iron, and arachidic acid contents in the substrate WS : OLPR 3 : 1 (v/v) (Figure 3), while its variation was most strongly correlated with magnesium, calcium, and iron and negatively correlated with total sugars, sucrose, fructose, glucose, manganese, potassium, and stearic and linolenic acid contents (Figure 4(d)). Mushroom oleic acid covaried with total carbohydrates, total protein, crude fiber, and oleic and linoleic acids in the substrate WS : OLPR 1 : 3 (v/v) (Figure 3) and was positively affected by total protein, total carbohydrates, and crude fiber contents and negatively with fat content (Figure 4(e)). Mushroom linoleic acid was positively correlated with palmitic and stearic acids, manganese, potassium, sodium, and sugar contents and negatively correlated with oleic and linoleic acids (Figure 4(f)). Mushroom linolenic acid the most positively correlated with fat content and negatively correlated with total protein, total carbohydrates, crude fiber, and linolenic acid contents (Figure 4(g)).

## 4. Discussion

Oyster mushroom could help managing the disposal of agrowastes [42], especially lignocellulosic ones [43], reducing environmental and soil pollutions [44, 45]. Production of the wheat straw substrate was earlier compared with that of the work of Naim et al. [46].

The substrate WS : OLPR 1 : 3 has hastened the fruit formation by 3.7 d in comparison with control, compared with a hastening by 3.4 d with WS : OLPR 3 : 1 substrate as reported by Koutrotsios et al. [47]. A similar trend was reported when a similar proportion of olive mill waste was added to wheat straw [48]. The WS : OLPR 1 : 3 substrate was initially richer in carbohydrates, basic foodstuffs for the mushroom nutrition [49], and proteins, as important source of nitrogen favoring the mushroom growth [50]. The earliness obtained in this substrate may be attributed to its higher initial lignin content compared with others. At the primary growth phase, assigned as primary metabolism, white-rot fungi degrade high-digestible polysaccharides into low-weight molecules and consume lignin and hemicellulose relatively more than cellulose [51]. Lignin degradation enables access to holocellulose, which is the carbon and energy source for this species [52]. On the contrary, at the secondary growth phase or, namely, secondary metabolism, these fungi intend to degrade cellulose more than hemicellulose and lignin [53]. Superiority in yield obtained in the wheat straw substrate may be because of the higher cellulose and hemicellulose contents initially, which provide a more easily accessible source of energy to the mycelia run. Moreover, hemicellulose, cellulose, and lignin contain carbon, hydrogen, and oxygen and serve as an energy source for fungal growth, a fact that explains their reduction along the cultivation cycle [54]. Since the conversion of lignocellulosic biomass into soluble sugars depends mainly on the production of various efficient lignocellulosic enzymes [55], it is imperative to test the extracellular enzymes secreted by the mycelium throughout its growth to complement the current findings.

Moreover, the biological efficiency obtained in wheat straw in the current study was higher than that reported in the earlier works [46, 55, 56]. Also, supplementation with maize waste water [19] and olive mill waste [48] resulted in lower biological efficiency of *P. ostreatus* in comparison with that obtained in the commercial control of the current study. Mushrooms obtained in all treatments were marketable showing a high PD/SL ratio. They had lower weight, comparable pileus diameter, and higher stipe length than those reported by Tesfay et al. [57]. In addition, mushrooms obtained in the wheat straw substrate had similar pileus diameter, lower stipe diameter, and higher stipe length in comparison to early findings of Girmay et al. [31]. Smaller mushroom size was reported by Kimenju et al. [55] on wheat straw substrate.

The incorporation of olive pruning residues in the growing substrate has ameliorated the mushroom nutritional value. The use of WS : OLPR 1 : 3 has caused an increase in protein, carbohydrates, crude fiber, and iron contents in mushrooms, while that of WS : OLPR 3 : 1 (v/v) and WS : OLPR 1 : 3 (v/v) substrates has caused a decrease in fat and sodium contents. The latter had lower moisture, carbohydrates, and crude fiber contents than those in other early studies on various substrate types [2, 58]. The reduction in carbohydrate contents of mushrooms was similarly reported by Koutrotsios et al. [47] on mixtures of wheat straw and olive pruning substrates. Mushrooms produced by wheat straw substrate of the present study had lower moisture, carbohydrates, protein, fat, crude fiber, calcium, iron, potassium, and higher sodium contents in comparison with results of Patil et al. [17], where various agrowastes were used as substrates for *P. ostreatus*.

Diets rich in fiber decrease the incidence of several diseases [59], and low sodium content helps controlling the blood pressure [60]. Early studies indicated four most preponderant fatty acids in oyster mushroom: linoleic, palmitic, oleic, and stearic acids [61, 62]. Around 89.5% of total fatty acids consisted of linoleic, palmitic, and oleic acids [63]. In the current study, the latter three fatty acids were the most abundant in tested mushrooms, ranging between 92.4 and 98.9% in the different substrates. Mushrooms fatty acids are divided into three groups: polyunsaturated, monounsaturated, and saturated with a dominance of unsaturated ones [3]. The ratio of polyunsaturated to saturated fatty acids (PUFA/SFA) was the highest in mushrooms grown on wheat straw (8.1), followed by those of mushrooms grown on WS : OLPR 3 : 1 (v/v) (3.7) and WS : OLPR 1 : 3 (v/v) (3.2) substrates, indicating an increase of saturated fatty acids content in mushrooms, as a result of olive pruning residues incorporation in the growing substrate. Mushrooms of the later were poorer in polyunsaturated fatty acids and richer in monounsaturated ones, compared with control mushrooms. Furthermore, all mushrooms by the different substrates had a higher PUFA/SFA ratio compared with those of the previous studies [61, 62]. Noting that foods with relative high PUFA/SFA ratio are considered healthy and of a strong hypocholesterolemic effect [64]. In terms of human dietary intakes, monounsaturated fatty acids help reducing the LDL level and polyunsaturated fatty acids reduce the risks of diabetes and coronary heart disease [65]. The richness of mushrooms in unsaturated fatty acids is the reason behind their use for medical purposes [66].

Furthermore, as the intake and synthesis of fatty acids from the substrate were not addressed in the literature, mushrooms may have obtained the palmitoleic acid from the wheat straw substrate; for this, fatty acid was not initially present in the substrates containing OLPR. Mushrooms may synthesize the myristic acid indirectly from substrates, as it was initially absent in all substrates types but detected in mushrooms produced in substrates containing OLPR. The strong correlation between oleic acid content in mushrooms with that of substrates suggests a direct uptake of this fatty acid, discarding the mushroom's need to synthesize it or to obtain it through conversion reactions. Moreover, the presence of arachidic acid in substrates containing OLPR and its absence in mushrooms may suggest its complete conversion into other fatty acids by the oyster mushroom.

## 5. Conclusion

The valorization of OLPR by their bioconversion into a nutritious food has been evidenced in the present study. Farmers may benefit from their use in low proportion by producing enough yields from a lower number of harvests. The main advantage of using this underestimated agricultural by-product is in the improvement of the nutritional value of mushrooms. Results suggest the potential use of OLPR as a nutritious supplement for the commercial wheat straw substrate.

## Figures and Tables

**Table 1:** Physicochemical properties of substrates..

	WS	OLPR	WS : OLPR 1 : 3 (v/v)	WS : OLPR 3 : 1 (v/v)
pH	5.5	6.3	7.4	7.0
EC (ms/cm)	0.3	1.3	1	0.4
OM (%)	86.6	92.2	90.9	92.0
C/N ratio	69 : 1	44 : 1	60 : 1	74 : 1

WS: wheat straw, OLPR: olive pruning residues, EC: electroconductivity, OM: organic matter, C: carbon, N: nitrogen, WS: wheat straw, OLPR: olive pruning residues, EC: electroconductivity, OM: organic matter, C: carbon, N: nitrogen.

**Table 2:** Substrate effect on various parameters of mushroom growth and production.

Treatment	100% MC (DAS)	FN	HF1 (DAS)	MW (g)	BY (g/bag)	BE (%)	EY (g/bag)
WS	7.7 ± 1.1^a^	3.0 ± 0.0^c^	34.7 ± 4.6^a^	13.1 ± 2.8^a^	910.1 ± 236.3^b^	105.0 ± 27.2^b^	871.4 ± 238.4^b^
WS : OLPR 3 : 1 (v/v)	7.7 ± 3.0^a^	2.0 ± 0.0^b^	46.0 ± 8.0^b^	12.2 ± 2.4^a^	624.9 ± 222.1^ab^	80.3 ± 28.5^ab^	590.0 ± 231.63^ab^
WS : OLPR 1 : 3 (v/v)	7.7 ± 0.6^a^	1.3 ± 0.6^a^	31.0 ± 1.0^a^	13.2 ± 3.5^a^	406.8 ± 11.6^a^	54.1 ± 1.5^a^	388.4 ± 11.82^a^
*p*value	1.00	0.00	0.03	0.89	0.04	0.09	0.05

Values are mean ± SD; means within the same column followed by the same letters are not significantly different at *p* < 0.05 according to Duncan's multiple range test. MC: mycelial colonization, FN: flush number, HF1: time to harvest the first flush, MW: individual mushroom weight, BY: biological yield, BE: biological efficiency, EY: economic yield, and DAS: days after spawning.

**Table 3:** Digestibility of fiber fraction (% dry weight) components.

	WS		WS : OLPR 3 : 1 (v/v)		WS : OLPR 1 : 3 (v/v)	
	I	R	I	R	I	R
Hemicellulose	21.36 ± 0.0^c^	0.6 ± 0.0^a^	13.47 ± 0.0^b^	2.42 ± 0.0^b^	6.73 ± 0.0^a^	3.38 ± 0.0^c^
Cellulose	41.05 ± 0.0^c^	27.34 ± 0.0^c^	37.35 ± 0.0^b^	19.49 ± 0.0^b^	22.62 ± 0.0^a^	12.74 ± 0.0^a^
Lignin	7.27 ± 0.0^a^	3.38 ± 0.0^a^	9.30 ± 0.0^b^	8.99 ± 0.0^b^	19.34 ± 0.0^c^	13.36 ± 0.0^c^
*p* value	0.00	0.00	0.00	0.00	0.00	0.00

WS: wheat straw, OLPR: olive pruning residues, I: initial substrate, and R: residual. Values are mean ± SD. Means within the same column followed by the same letters of lowercase (corresponding to initial substrate: I) or uppercase (corresponding to residual substrate: R) are not significantly different at *p* < 0.05 according to Duncan's multiple range test.

**Table 4:** Mushroom (% fresh weight) and substrate (% dry weight) composition.

	WS		WS : OLPR 3 : 1 (v/v)		WS : OLPR 1 : 3 (v/v)		*p*_value_	
	S	M	S	M	S	M	S	M
Moisture	12.8 ± 0.2^a^	87.7 ± 0.8^A^	20.0 ± 0.2^b^	87.5 ± 0.9^A^	28.7 ± 0.1^c^	88.0 ± 0.4^A^	0.00	0.57
Total protein	5.5 ± 0.0^b^	2.9 ± 0.0^B^	5.00 ± 0.1^a^	3.7 ± 0.0^C^	7.3 ± 0.0^c^	2.3 ± 0.0^A^	0.00	0.00
Crude fiber	38.4 ± 0.0^a^	3.9 ± 0.0^B^	38.5 ± 0.0^a^	5.3 ± 0.0^C^	40.0 ± 0.0^b^	2.9 ± 0.0^A^	0.00	0.00
Carbohydrates	38.5 ± 0.0^a^	6.2 ± 0.0^B^	38.6 ± 0.0^a^	6.3 ± 0.0^C^	40.1 ± 0.0^b^	5.0 ± 0.0^A^	0.00	0.00
Fat	0.7 ± 0.0^b^	0.3 ± 0.0^B^	1.04 ± 0.00^c^	0.11 ± 0.00^A^	0.53 ± 0.00^a^	0.11 ± 0.00^A^	0.00	0.00
TSS	1.5 ± 0.0^c^	0.01 ± 0.00^A^	0.16 ± 0.00^a^	0.10 ± 0.00^B^	0.37 ± 0.00^b^	0.11 ± 0.00^C^	0.00	0.00
Fructose	0.6 ± 0.0^c^	0.01 ± 0.00^A^	0.01 ± 0.00^a^	0.01 ± 0.02^A^	0.04 ± 0.00^b^	0.01 ± 0.00^A^	0.00	0.21
Glucose	0.7 ± 0.0^c^	0.01 ± 0.00^A^	0.15 ± 0.00^a^	0.09 ± 0.01^B^	0.33 ± 0.00^b^	0.1 ± 0.0^B^	0.00	0.00
Sucrose	0.2 ± 0.0^b^	<0.005^A^	<0.005^a^	<0.005^A^	<0.005^a^	<0.005^A^	0.00	—

Values are mean ± SD; means within the same column followed by the same letters of lowercase (corresponding to substrate: S) or uppercase (corresponding to mushroom: M) are not significantly different at *p* < 0.05 according to Duncan's multiple range test. WS: wheat straw, OLPR: olive pruning residues, S: substrate, M: mushroom, and TSS: total soluble sugar.

**Table 5:** Mineral composition (% dry weight) of substrates and mushrooms.

	WS		WS : OLPR 3 : 1 (v/v)		WS : OLPR 1 : 3 (v/v)		*p*_value_	
	S	M	S	M	S	M	S	M
Ca	0.42 ± 0.00^a^	0.003 ± 0.000^A^	0.89 ± 0.00^b^	0.003 ± 0.000^A^	0.93 ± 0.002^c^	0.003 ± 0.000^A^	0.00	0.43
K	1.43 ± 0.01^d^	0.25 ± 0.00^A^	0.88 ± 0.00^b^	0.32 ± 0.00^C^	0.95 ± 0.002^c^	0.27 ± 0.001^B^	0.00	0.00
Mn	0.08 ± 0.00^c^	0.00003 ± 0.000^A^	0.004 ± 0.001^a^	0.00006 ± 0.000^A^	0.004 ± 0.001^a^	0.0001 ± 0.000^A^	0.00	0.28
Fe	0.015 ± 0.002^a^	0.002 ± 0.000^A^	0.19 ± 0.00^c^	0.003 ± 0.000^B^	0.17 ± 0.002^b^	0.002 ± 0.000^A^	0.00	0.00
Na	0.068 ± 0.003^c^	0.42 ± 0.00^B^	0.063 ± 0.002^b^	0.007 ± 0.001^A^	0.060 ± 0.003^b^	0.006 ± 0.001^A^	0.03	0.00
Mg	0.083 ± 0.003^a^	0.134 ± 0.002^A^	0.140 ± 0.004^b^	0.147 ± 0.001^A^	0.140 ± 0.002^b^	0.147 ± 0.002^A^	0.00	0.65

Values are mean ± SD; means within the same column followed by the same letters of lowercase (corresponding to substrate: S) or uppercase (corresponding to mushroom: M) are not significantly different at *p* < 0.05 according to Duncan's multiple range test, S: substrate; M: mushroom.

**Table 6:** Fatty acid profile of substrates and mushrooms (dry basis, % of total fatty acids).

	WS		WS : OLPR 3 : 1 (v/v)		WS : OLPR 1 : 3 (v/v)		*p*_value_	
	S	M	S	M	S	M	S	M
*PUFA*								
C18 : 2 linoleic acid	21.06 ± 0.003^a^	80.58 ± 0.002^C^	32.77 ± 0.002^b^	68.46 ± 0.002^B^	49.18 ± 0.002^c^	64.24 ± 0.002^A^	0.00	0.00
C18 : 3 linolenic acid	8.06 ± 0.001^c^	0.20 ± 0.001^A^	3.08 ± 0.002^a^	0.46 ± 0.001^B^	6.76 ± 0.003^b^	Nd	0.00	0.00

*MUFA*								
C16 : 1 palmitoleic acid	1.20 ± 0.004^a^	0.09 ± 0.004^A^	Nd	4.27 ± 0.002^C^	Nd	1.24 ± 0.002^B^	0.00	0.00
C18 : 1 oleic acid	10.48 ± 0.002^a^	9.10 ± 0.002^A^	13.57 ± 0.002^b^	9.10 ± 0.000^A^	17.40 ± 0.002^c^	14.70 ± 0.002^B^	0.00	0.00

*SFA*								
C20 : 0 arachidic acid	Nd	Nd	5.17 ± 0.001^b^	nd	2.26 ± 0.002^a^	Nd	0.00	0.00
C16 : 0 palmitic acid	53.32 ± 0.002^c^	9.18 ± 0.001^A^	40.62 ± 0.002^b^	14.86 ± 0.002^B^	19.60 ± 0.002^a^	16.44 ± 0.002^C^	0.00	0.00
C18 : 0 stearic acid	5.88 ± 0.001^c^	0.84 ± 0.002^A^	4.78 ± 0.002^a^	3.22 ± 0.004^C^	4.80 ± 0.002^b^	3.17 ± 0.002^B^	0.00	0.00
C14 : 0 myristic acid	nd	Nd	Nd	0.54 ± 0.001^B^	Nd	0.20 ± 0.002^A^	0.00	0.00

Values are mean ± SD; means within the same column followed by the same letters of lowercase (corresponding to substrate: S) or uppercase (corresponding to mushroom: M) are not significantly different at *p* < 0.05 according to Duncan's multiple range test. PUFA: polyunsaturated fatty acids, MUFA: monounsaturated fatty acids, SFA: saturated fatty acids, S: substrate, M: mushroom, and nd: not detected.

**Table 7:** Overview of PLSR model for mushroom fatty acids in relation to substrate composition.

		*q* ^*2*^ cum		*r* ^2^ cum	
Mushroom fatty acid	*RMSE*	Comp 1	Comp 2	Comp 1	Comp 2
C14 : 0 myristic acid	0.007	0.494	0.990	0.588	0.997
C16 : 0 palmitic acid	0.158	0.973	0.997		
C18 : 1 oleic acid	0.216	0.108	0.984		
C18 : 3 linolenic acid	0.013	0.112	0.981		
C16 : 1 palmitoleic acid	0.063	0.372	0.988		
C18 : 0 stearic acid	0.044	0.986	0.997		
C18 : 2 linoleic acid	0.356	0.958	0.997		

*RMSE:* root-mean squared-error of calibration model and prediction model, respectively; comp: component.

## Data Availability

The data used to support the findings of this study have not been made available because it is a part of a PhD study.

## References

[1] Bellettini M. B., Fiorda F. A., Maieves H. A. (2019). Factors affecting mushroom Pleurotus spp. *Saudi Journal of Biological Sciences*.

[2] Tolera K. D., Abera S. (2017). Nutritional quality of oyster mushroom (*Pleurotus ostreatus*) as affected by osmotic pretreatments and drying methods. *Food Science & Nutrition*.

[3] Senatore F., Dini A., Marino A., Schettino O. (1988). Chemical constituents of some basidiomycetes. *Journal of the Science of Food and Agriculture*.

[4] Croan S. C. (2004). Conversion of conifer wastes into edible and medicinal mushrooms. *Forest Products Journal*.

[5] Vamanu E. (2012). Biological activities of the polysaccharides produced in submerged culture of two edible Pleurotus ostreatus mushrooms. *Journal of Biomedicine and Biotechnology*.

[6] Yang Z., Xu J., Fu Q. (2013). Antitumor activity of a polysaccharide from *Pleurotus eryngii* on mice bearing renal cancer. *Carbohydrate Polymers*.

[7] Philippoussis A., Zervakis G., Diamantopoulou P. (2001). Bioconversion of agricultural ligno-cellulosic wastes through the cultivation of the edible mushrooms *Agrocybe aegerita, Volvariella volvaceae* and *Pleurotus* spp.. *World Journal of Microbiology and Biotechnology*.

[8] Olivieri G., Marzocchella A., Salatino P., Giardina P., Cennamo G., Sannia G. (2006). Olive mill wastewater remediation by means of *Pleurotus ostreatus*. *Biochemical Engineering Journal*.

[9] Kong W. S. (2004). Spawn, descriptions of commercially important Pleurotus species, part II. Oyster mushroom. *Mushroom Growers’ Handbook 1: Oyster Mushroom Cultivation, MushWorld*.

[10] Hoa H. T., Wang C.-L., Wang C.-H. (2015). The effects of different substrates on the growth, yield, and nutritional composition of two oyster mushrooms (Pleurotus ostreatus and Pleurotus cystidiosus). *Mycobiology*.

[11] Goyal R., Grewal R. B., Goyal R. K. (2006). Nutritional attributes of *Agaricus bisporus* and *Pleurotus sajor caju* mushrooms. *Nutrition and Health*.

[12] Gothwal R., Gupta A., Kumar A., Sharma S., Alappat B. J. (2012). Feasibility of dairy waste water (DWW) and distillery spent wash (DSW) effluents in increasing the yield potential of *Pleurotus flabellatus* (PF 1832) and *Pleurotus sajor-caju* (PS 1610) on bagasse. *Biotechnology*.

[13] Oyetayo O. V., Ariyo O. O. (2013). Micro and macronutrient properties of Pleurotus ostreatus (jacq: fries) cultivated on different wood substrates. *Jordan Journal of Biological Sciences*.

[14] El Sebaaly Z., Assadi F., Sassine Y. N., Shaban N. (2019). Substrate types effect on nutritional composition of button mushroom (*Agaricus bisporus*). *Agriculture and Forestry*.

[15] Koutrotsios G., Mountzouris K. C., Chatzipavlidis I., Zervakis G. I. (2014). Bioconversion of lignocellulosic residues by Agrocybe cylindracea and Pleurotus ostreatus mushroom fungi-assessment of their effect on the final product and spent substrate properties. *Food Chemistry*.

[16] Koutrotsios G., Kalogeropoulos N., Kaliora A. C., Zervakis G. I. (2018). Toward an increased functionality in oyster (Pleurotus) mushrooms produced on grape marc or olive mill wastes serving as sources of bioactive compounds. *Journal of Agricultural and Food Chemistry*.

[17] Patil S. S., Ahmed S. A., Telang S. M., Baig M. M. V. (2010). The nutritional value of *Pleurotus ostreatus* (Jacq.:Fr.) kumm cultivated on different lignocellulosic agrowastes. *Innovative Romanian Food Biotechnology*.

[18] Labuschagne P. M., Eicker A., Aveling T. A. S., de Meillon S., Smith M. F. (2000). Influence of wheat cultivars on straw quality and Pleurotus ostreatus cultivation. *Bioresource Technology*.

[19] Loss E., Royer A., Rodrigues M., Barana A. C. (2009). Use of maize wastewater for the cultivation of the *Pleurotus* spp. mushroom and optimization of its biological efficiency. *Journal of Hazardous Materials*.

[20] Zervakis G. I., Koutrotsios G., Katsaris P. (2013). Composted versus raw olive mill waste as substrates for the production of medicinal mushrooms: an assessment of selected cultivation and quality parameters. *BioMed Research International*.

[21] Michael H. W., Bultosa G., Pant L. M. (2011). Nutritional contents of three edible oyster mushrooms grown on two substrates at Haramaya, Ethiopia, and sensory properties of boiled mushroom and mushroom sauce. *International Journal of Food Science & Technology*.

[22] USDA (United States Department of Agriculture)-Soil Conservation Service (1996). Chapter 4 Agricultural waste characteristics. *Agriculture Waste Management Field Handbook*.

[23] Obi F., Ugwuishiwu B., Nwakaire J. (2016). Agricultural waste concept, generation, utilization and management. *Nigerian Journal of Technology*.

[24] El Sebaaly Z., Abou Fayssal S., Shaban N., Sassine Y. N. Growing Agaricus bisporus on compost mixtures based on chicken manure and banana residues.

[25] Sagani A., Hagidimitriou M., Dedoussis V. A study of burning olive tree pruning biomass for electricity generation.

[26] Ghoneim A. M., Elbassir O. I., Modahish A. S., Mahjoub M. O. (2016). Compost production from olive tree pruning wastes enriched with phosphate rock. *Compost Sciences and Utilization*.

[27] Wang W., Tai F., Hu X. (2010). Current initiatives in proteomics of the olive tree. *Olives and Olive Oil in Health and Disease Prevention*.

[28] Muzzalupo I., Micali S., Muzzalupo I., Micali S. (2015). *Agricultural and Food Biotechnologies of Olea Europaea and Stone Fruits*.

[29] Lebanese Ministry of Agriculture (MoA) and Food and Agriculture Organization (FAO) (2010). *The Core Module of the Census of Agriculture*.

[30] Pardo-Giménez A., Zied D. C., Picornell Buendía M. R., de Juan Valero J. A., PardoGonzález J. E. Cultivation of *Pleurotus ostreatus* using supplemented spent oyster mushroom substrate.

[31] Girmay Z., Gorems W., Birhanu G., Zewdie S. (2016). Growth and yield performance of *Pleurotus ostreatus* (Jacq. Fr.) Kumm (oyster mushroom) on different substrates. *AMB Express*.

[32] Carvalho C. S. M. d., Aguiar L. V. B. d., Sales-Campos C., Minhoni M. T. d. A., Andrade M. C. N. d. (2012). Applicability of the use of waste from different banana cultivars for the cultivation of the oyster mushroom. *Brazilian Journal of Microbiology*.

[33] Association of Official Chemistry–AOAC (1984). *Official Methods of Analysis of the Association Of Official Analytical Chemists*.

[34] Association of Official Chemistry–AOAC (1990). *Official Methods of Analysis of the Association of Official Analytical Chemists*.

[35] Ajlouni S. O., Beelman R. B., Thompson D. B., Mau J. L., Charalambous G. (1995). Changes in soluble sugars in various tissues of cultivated mushrooms, *Agaricus bisporus* during postharvest storage. *Food Favors*.

[36] Nieto I. J., Chegwin C. (2013). The effect of different substrates on triterpenoids and fatty acids in fungi of the genus *Pleurotus*. *Journal of the Chilean Chemical Society*.

[37] Association of Official Analytical Chemists–AOAC (1995). *Official Methods of Analysis of the Association of Official Analytical Chemists*.

[38] Dreywood R. (1946). Qualitative test for carbohydrate material. *Industrial & Engineering Chemistry Analytical Edition*.

[39] Reis F. S., Barros L., Martins A., Ferreira I. C. F. R. (2012). Chemical composition and nutritional value of the most widely appreciated cultivated mushrooms: an inter-species comparative study. *Food and Chemical Toxicology*.

[40] Akata I., Ergonul P. G., Ergonul B., Kalyoncu F. (2013). Determination of fatty acid contents of five wild edible mushroom species collected from Anatolia. *Journal of Pure and Applied Microbiology*.

[41] Zhang X., Cheng Z., Ma L., Li J. (2017). A study on accumulation of volatile organic compounds during ochratoxin a biosynthesis and characterization of the correlation in *Aspergillus carbonarius* isolated from grape and dried vine fruit. *Food Chemistry*.

[42] Das N., Mukherjee M. (2007). Cultivation of Pleurotus ostreatus on weed plants. *Bioresource Technology*.

[43] Mandeel Q. A., Al-Laith A. A., Mohamed S. A. (2005). Cultivation of oyster mushrooms (Pleurotus spp.) on various lignocellulosic wastes. *World Journal of Microbiology and Biotechnology*.

[44] Beetz A., Kustudia M. (2004). *Mushroom Cultivation and Marketing. Horticulture Production Guide*.

[45] Kapahi M., Sachdeva S. (2017). Mycoremediation potential of *Pleurotus* species for heavy metals: a review. *Bioresources and Bioprocessing*.

[46] Naim L., Alsanad M. A., El Sebaaly Z., Shaban N., Fayssal S. A., Sassine Y. N. (2020). Variation of Pleurotus ostreatus (Jacq. Ex Fr.) P. Kumm. (1871) performance subjected to differentdoses and timings of nano-urea. *Saudi Journal of Biological Sciences*.

[47] Koutrotsios G., Patsou M., Mitsou E. K. (2019). Valorization of olive by-products as substrates for the cultivation of ganoderma lucidum and Pleurotus ostreatus mushrooms with enhanced functional and prebiotic properties. *Catalysts*.

[48] Ruiz-Rodriguez A., Soler-Rivas C., Polonia I., Wichers H. J. (2010). Effect of olive mill waste (OMW) supplementation to Oyster mushrooms substrates on the cultivation parameters and fruiting bodies quality. *International Biodeterioration & Biodegradation*.

[49] Stamets P., Chilton J. S. (1983). *The Mushroom Cultivator. A Practical Guide to Growing Mushrooms at Home*.

[50] Zied D. C., Savoie J. M., Pardo-Giménez A. P., El-Shemy H. (2011). Soybean the main nitrogen source in cultivation substrates of edible and medicinal mushrooms. *Soybean and nutrition*.

[51] Zadrazil F., Kamra D. N., Isikhuemhen O. S., Schuchardt F., Flachowsky G. (1996). Bioconversion of lignocellulose into ruminant feed with white rot fungi-review of work done at the FAL, braunschweig. *Journal of Applied Animal Research*.

[52] Xiao Q., Ma F., Li Y., Yu H., Li C., Zhang X. (2017). Differential proteomic profiles of *Pleurotus ostreatus* in response to lignocellulosic components provide insights into divergent adaptive mechanisms. *Frontiers in Microbiology*.

[53] Andrade M. C. N. d., Minhoni M. T. d. A., Sansígolo C. A., Zied D. C. (2010). Análise química da madeira e casca de diferentes tipos de eucalipto antes e durante o cultivo de shiitake em toras. *Revista Árvore*.

[54] Maki M., Leung K. T., Qin W. (2009). The prospects of cellulase-producing bacteria for the bioconversion of lignocellulosic biomass. *International Journal of Biological Sciences*.

[55] Kimenju J. W., Odero G. O. M., Mutitu E. W., Wachira P. M., Narla R. D., Muiru W. M. (2009). Suitability of local available substrates for oyster mushroom (*Pleurotus ostreatus*) cultivation in Kenya. *Asian Journal of Plant Sciences*.

[56] Mejía S. J., Albertó E. (2013). Heat treatment of wheat straw by immersion in hot water decreases mushroom yield in *Pleurotus ostreatus*. *Revista Iberoamericana de Micología*.

[57] Tesfay T., Godifey T., Mesfin R., Kalayu G. (2020). Evaluation of waste paper for cultivation of oyster mushroom (*Pleurotus ostreatus*) with some added supplementary materials. *AMB Express*.

[58] Jin Z., Li Y., Ren J., Qin N. (2018). Yield, nutritional content, and antioxidant activity ofPleurotus ostreatuson corncobs supplemented with herb residues. *Mycobiology*.

[59] Dhingra D., Michael M., Rajput H., Patil R. T. (2012). Dietary fibre in foods: a review. *Journal of Food Science and Technology*.

[60] Lister C. E. (2015). Nutritional analysis of mushrooms-a summary.

[61] Ergönül P. G., Akata I., Kalyoncu F., Ergönül B. (2013). Fatty acid compositions of six wild edible mushroom species. *The Scientific World Journal*.

[62] Bengu A. S. (2020). The fatty acid composition in some economic and wild edible mushrooms in Turkey. *Progress in Nutrition*.

[63] Pedneault K., Angers P., Avis T. J., Gosselin A., Tweddell R. J. (2007). Fatty acid profiles of polar and non-polar lipids of Pleurotus ostreatus and P. cornucopiae var. “citrino-pileatus” grown at different temperatures. *Mycological Research*.

[64] Chang N. W., Huang P. C. (1998). Effects of the ratio of polyunsaturated and monounsaturated fatty acid to saturated fatty acid on rat plasma and liver lipid concentrations. *Lipids*.

[65] Food and Agriculture Organization of the United Nations (2008). *Fats and Fatty Acids in Human Nutrition*.

[66] Türkekul I., Yılmaz N., Şahin F., Bayrak Ö.F. (2010). Fatty acid composition of six mushroom samples of black sea region of Turkey. *Asian Journal of Chemistry*.

